# Pecularities and applications of aryl-alcohol oxidases from fungi

**DOI:** 10.1007/s00253-021-11337-4

**Published:** 2021-05-17

**Authors:** Vlada B. Urlacher, Katja Koschorreck

**Affiliations:** Institute of Biochemistry, Heinrich-Heine-University Düsseldorf, Universitätsstraße 1, 40225 Düsseldorf, Germany

**Keywords:** Aryl-alcohol oxidase, GMC family, Lignin degradation, Aryl alcohols, Aliphatic allylic alcohols, Biocatalysis, Bio-based precursors, Flavors

## Abstract

**Abstract:**

Aryl-alcohol oxidases (AAOs) are FAD-containing enzymes that oxidize a broad range of aromatic as well as aliphatic allylic alcohols to aldehydes. Their broad substrate spectrum accompanied by the only need for molecular oxygen as cosubstrate and production of hydrogen peroxide as sole by-product makes these enzymes very promising biocatalysts. AAOs were used in the synthesis of flavors, fragrances, and other high-value-added compounds and building blocks as well as in dye decolorization and pulp biobleaching. Furthermore, AAOs offer a huge potential as efficient suppliers of hydrogen peroxide for peroxidase- and peroxygenase-catalyzed reactions. A prerequisite for application as biocatalysts at larger scale is the production of AAOs in sufficient amounts. Heterologous expression of these predominantly fungal enzymes is, however, quite challenging. This review summarizes different approaches aiming at enhancing heterologous expression of AAOs and gives an update on substrates accepted by these promising enzymes as well as potential fields of their application.

**Key points:**

• *Aryl-alcohol oxidases (AAOs) supply ligninolytic peroxidases with H*_*2*_*O*_*2*_.

• *AAOs accept a broad spectrum of aromatic and aliphatic allylic alcohols.*

• *AAOs are potential biocatalysts for the production of high-value-added bio-based chemicals.*

## Introduction

**Fig. 1:**
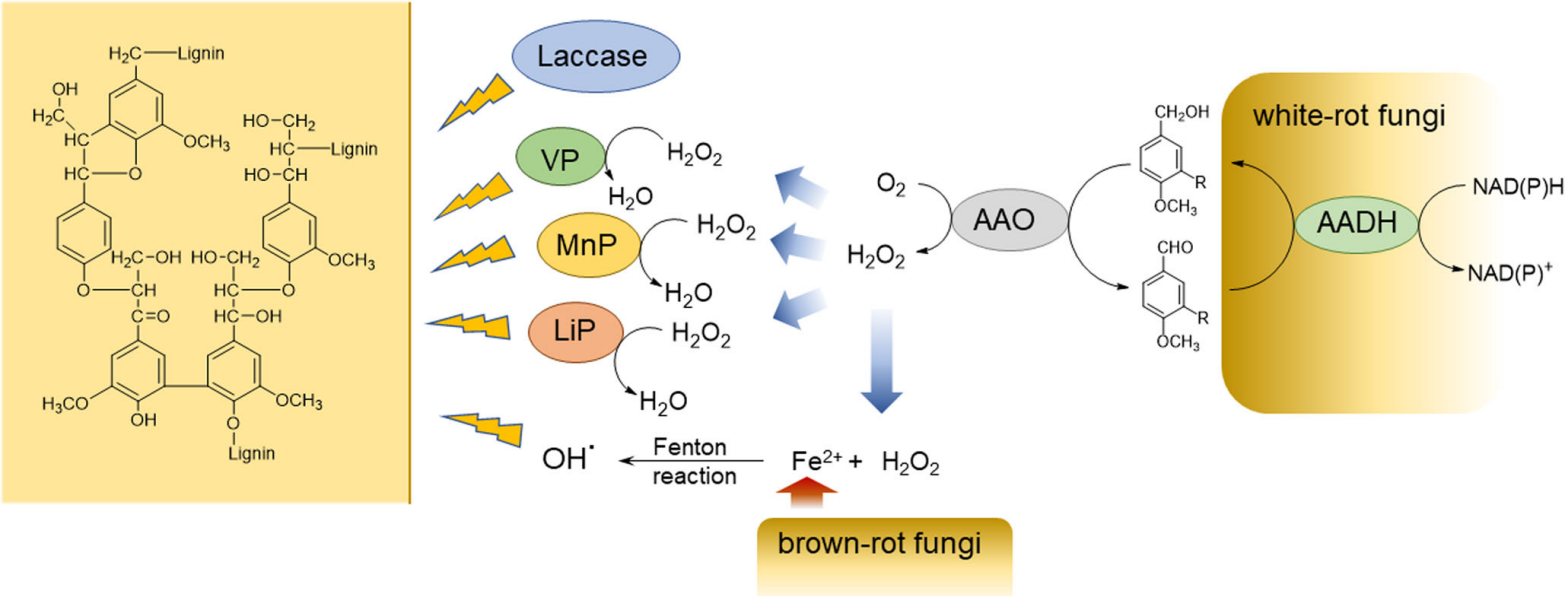
Involvement of AAO in lignin degradation. *VP* versatile peroxidase, *MnP* manganese peroxidase, *LiP* lignin peroxidase, *AADH* aryl-alcohol dehydrogenase

Since decades, oxidoreductases have been recognized as valuable tools for synthetic chemistry, because they catalyze reactions that are often difficult or impossible to achieve with classical chemical catalysts (Hall 2020). Oxidases, reductases, oxygenases, and dehydrogenases that enable selective synthesis of chiral alcohols, ketones, aldehydes, and carbon acids have been successfully integrated into biocatalytic and chemo-enzymatic processes. Oxidoreductases are of vital interest also for developing the bioeconomy via efficient conversion of renewable feedstocks like lignocellulose to fuels and high-value-added chemicals. White-rot fungi secrete a bunch of oxidative enzymes participating in the degradation of lignocellulosic biomass as a central step in carbon recycling. Peroxidases responsible for lignin degradation require sources of H_2_O_2_ (Hammel 1997). Thus, fungi produce oxidases such as aryl-alcohol oxidases that generate H_2_O_2_ required by the peroxidases (Fig. 1). Aryl-alcohol oxidases (AAOs, EC 1.1.3.7), also known as veratryl-alcohol oxidases, aromatic alcohol oxidases, or benzyl-alcohol oxidases, are flavin-adenine-dinucleotide (FAD)–containing enzymes that catalyze the oxidation of aromatic and aliphatic allylic primary alcohols to the corresponding aldehydes while reducing molecular oxygen to H_2_O_2_ (Guillen et al. 1992). Some other flavin-containing oxidases like vanillyl alcohol oxidase (EC 1.1.3.38) or 4-hydroxymandelate oxidase (decarboxylating; EC 1.1.3.19) that can also oxidize aromatic or phenolic compounds (Bhat and Vaidyanathan 1976; de Jong et al. 1992b; Ewing et al. 2020; Martin et al. 2020) will not be considered herein.

Within this review, we will focus on AAOs from fungi. Since their discovery in 1960, significant knowledge has been gained with regard to structural, spectral, biochemical, and catalytic properties of fungal AAOs. All these achievements are a subject of several excellent reviews (Hernandez-Ortega et al. 2012a; Serrano et al. 2020) and therefore will only be touched briefly here. Like all oxidases, AAOs are independent of the costly nicotinamide cofactors required by oxygenases, and rely only on molecular oxygen as final electron acceptor. These features combined with the broad substrate spectrum and quite high stability make these enzymes potential biocatalysts for the production of bio-based precursors for plastics, bioactive compounds, and fragrances. Indeed, a number of publications, particularly those which have appeared in the last years, are dedicated to the use of AAOs in biocatalysis. Recently, progress was also made in protein engineering of AAOs for improved expression, enhanced activity, and increased selectivity. Broader exploitation and protein engineering of AAOs are however challenged by their difficult accessibility due to low concentrations in original fungi and insufficient expression in recombinant microorganisms. Whereas we will not specifically focus on the methods of enzyme engineering of AAOs described in a recent review (Vina-Gonzalez and Alcalde 2020), we will include a number of examples focusing on synthetic and biotechnological applications of wild-type AAOs and engineered variants thereof, and discuss various heterologous hosts and strategies aiming to enhance heterologous expression of these enzymes.

## Occurrence and GMC superfamily

Since the first report in 1960 by Farmer et al. (1960), who identified aromatic alcohol oxidase activity in the growth medium of the basidiomycete *Polystictus (Trametes) versicolor*, AAO activity has been found in cultures of many other basidiomycetes like *Pleurotus* species (Bourbonnais and Paice 1988; Galperin et al. 2016; Guillen et al. 1990; Sannia et al. 1991; Varela et al. 2000), *Bjerkandera adusta* (Muheim et al. 1990b; Romero et al. 2009), *Bjerkandera* sp. BOS55 (de Jong et al. 1992a), *Coprinopsis cinerea* (Tamaru et al. 2018), *Phanerochaete chrysosporium* (Asada et al. 1995), *Ustilago maydis* (Couturier et al. 2016), and ascomycetous fungi such as *Aspergillus terreus* (Chakraborty et al. 2014), *Botrytis cinerea* (Goetghebeur et al. 1992), *Fusarium proliferatum* (Regalado et al. 1999), *Geotrichum candidum* (Kim et al. 2001), and *Thermothelomyces thermophilus* (Kadowaki et al. 2020)*.* Although predominantly found in fungi, enzymes with AAO activities have also been reported in bacteria *Sphingobacterium* sp. (Tamboli et al. 2011), *Streptomyces* sp. (Riyadi et al. 2020), and *Methylovorus* sp. (Dijkman and Fraaije 2014), in the phytopathogenic insects *Chrysomela populi* and *Phratora vitellinae* (Brückmann et al. 2002), and in gastropods such as *Arion ater*, *Helix aspersa*, and *Limax flavus* (Large and Connock 1993; Mann et al. 1989).

According to their sequence and structure similarity, most flavin-containing oxidases have been classified in six families (Dijkman et al. 2013; Martin et al. 2020). AAOs belong to one of these families, the glucose-methanol-choline (GMC) oxidoreductase family, defined by Cavener in 1992 (Cavener 1992). GMC oxidoreductases were identified in ascomycetous and basidiomycetous fungi, plants, insects, and bacteria (Cavener 1992; Iida et al. 2007; Martin et al. 2020). Recently, hydroxymethylfurfural oxidases (HMFOs; EC 1.1.3.47) have been discovered which also belong to the GMC oxidoreductase family and oxidize aromatic primary alcohols, aldehydes, and thiols (Dijkman and Fraaije 2014; Ewing et al. 2014; Vinambres et al. 2020). While AAOs are secreted by fungi, HMFOs have been found in bacteria like *Methylovorus* sp. strain MP688 and *Pseudomonas nitroreducens*. HMFO from *Methylovorus* sp. strain MP688 is, for example, an intracellular bacterial enzyme with low sequence identity to AAOs (Dijkman and Fraaije 2014).

In 2013, the GMC oxidoreductases were assigned to the “Auxiliary Activities 3” (AA3) family in the Carbohydrate-Active enZyme (CAZy) database (Levasseur et al. 2013). The “auxiliary” enzymes help the carbohydrate-active enzymes like glycoside hydrolases, polysaccharide lyases, and carbohydrate esterases to gain a better access to carbohydrates in plant cell wall. Four subfamilies belong to the AA3 family: AA3_1 includes for the most part cellobiose dehydrogenases (CDHs, EC 1.1.99.18); AAOs together with glucose 1-oxidases (GOx, EC 1.1.3.4), glucose-1-dehydrogenases (GDHs, EC 1.1.5.9), and pyranose dehydrogenases (PDHs, EC 1.1.99.29) belong to the subfamily AA3_2; AA3_3 contains alcohol oxidases (AOx, EC 1.1.3.13); and AA3_4 comprises pyranose 2-oxidase (POx, EC 1.1.3.10).

The spectrum of substrates accepted by GMC oxidoreductases covers a broad range of sugars and alcohols. Depending on the type of the final electron acceptor, the GMC family members can be divided into two groups: oxidases, that transfer electrons to molecular oxygen, and dehydrogenases that transfer electrons to quinones, phenoxy radicals, or metal ions and show no or negligible oxygen reactivity. Fungal aryl-alcohol:quinone oxidoreductases (AAQOs) possess a similar substrate spectrum as AAOs but utilize quinones as final electron acceptor rather than molecular oxygen (Mathieu et al. 2016). Based on sequence similarities of ~10,000 putative fungal GMC oxidoreductases, these enzymes were classified in five clusters: AOx, CDH, POx, GOx–GDH, and AAO–PDH (Sützl et al. 2019). Phylogenetic and sequence studies of the individual clusters shed light into the sequence-structure-function relationship of the GMC family (Sützl et al. 2019). According to this analysis, the AAO-PDH cluster showed a split into three clades: AAO, AAO-like, and PDH. AAOs and AAQOs did not appear in separate clades, indicating that only minor amino acid changes determine the specificity for the final electron acceptor.

## Functions in nature

As mentioned above, in nature, AAOs are involved in degradation of lignocellulosic biomass. Lignocellulose is the most abundant renewable feedstock that provides a rich source for biofuels and value-added chemicals. Thus, effective bioconversion of lignocellulosic biomass has attracted significant attention from both academic and industrial points of view (Becker and Wittmann 2019). The most recalcitrant component of lignocellulose is lignin that can be decomposed by fungi and some bacterial species (Bugg et al. 2011; de Gonzalo et al. 2016). Wood-decaying white-rot fungi degrade lignin by using a large set of enzymes including lignin-modifying oxidative enzymes and “auxiliary” enzymes that do not degrade lignin on their own but assist other enzymes during lignin decomposition. Fungal oxidative enzymes involved in lignin degradation are laccases and the class II peroxidases lignin peroxidase (LiP), manganese peroxidase (MnP), and versatile peroxidase (VP) (Fig. 1). “Auxiliary” enzymes involved in lignin degradation include H_2_O_2_-generating AAO, glyoxal oxidase (GLOX, EC 1.2.3.5), and pyranose 2-oxidase but also CDH, GDH, and others. The well-studied lignin-degrading white-rot fungus *Pycnoporus cinnabarinus* contains, for example, 9 class II peroxidase encoding sequences, 5 putative laccase genes, 7 putative glyoxal oxidase genes, and 3 AAO encoding sequences (Levasseur et al. 2013). In comparison, brown-rot fungi lack class II peroxidases and instead produce enzymes that are involved in extracellular formation of Fenton’s reagent (Levasseur et al. 2014) (Fig. 1). Fe(II) reduces H_2_O_2_ to hydroxyl free radicals which cause cellulose degradation and methanol release from lignin by demethylation (Martinez et al. 2011). Methanol is used by methanol oxidase (MOX) to produce H_2_O_2_. MOX is the most abundant GMC oxidoreductase and the main oxidase involved in wood decay by brown-rot fungi (Daniel et al. 2007; Ferreira et al. 2015a). Thus, white-rot fungi rely on H_2_O_2_-producing enzymes like AAOs for ligninolytic peroxidases while in brown-rot fungi H_2_O_2_ released by MOX is utilized for Fenton’s reagent formation. This is in agreement with the higher number of AAOs found in peroxidase-producing white-rot fungi compared to brown-rot fungi (Ferreira et al. 2015a; Levasseur et al. 2013). Furthermore, extracellular production of AAO along with LiP by the white-rot fungus *B. adusta* (Muheim et al. 1990a) and AAO together with LiP and MnP by the white-rot fungi *P. ostreatus* and *T. pubescens* (Casieri et al. 2008) supports the hypothesis that AAOs are involved in lignin decomposition. Notably, the thermophilic cellulolytic ascomycete *Thermothelomyces thermophilus* M77 produces AAO but no lignin or manganese peroxidase (Berka et al. 2011), which suggests that AAO might be also involved in Fenton’s reagent formation.

To supply class II peroxidases with H_2_O_2_ for breakdown of lignin, wood-decaying fungi produce small aromatic aldehydes like anisaldehyde or veratraldehyde that are reduced by NADPH-dependent aryl-alcohol dehydrogenases to the corresponding alcohols which are accepted by AAOs as substrates (Guillen and Evans 1994). AAOs complete the redox cycle by transferring the electrons from aryl alcohols to molecular oxygen thus producing H_2_O_2_ and aldehydes. Lignin-derived aryl alcohols and aldehydes may also be used as substrates by AAOs for H_2_O_2_ production. Besides oxidizing benzylic alcohols produced by fungi or released in course of lignin decomposition, AAOs accept a broad range of other substrates, which are described in more detail in the following.

## Structure and mechanism

Members of the GMC family contain FAD as cofactor and share a similar topologic fold: They are composed of a highly conserved N-terminal domain involved in FAD binding, and a less conserved C-terminal domain involved in substrate binding (Martin et al. 2020). The N-terminal domain has the Rossmann super-secondary structure with an ADP-binding βαβ fold. The variability of the C-terminal domain reflects the diversity of substrates accepted by the members of this family (Sützl et al. 2018). Common to all GMC members is the presence of a highly conserved catalytic histidine residue involved in substrate oxidation and FAD reoxidation (Martin et al. 2020).

**Fig. 2:**
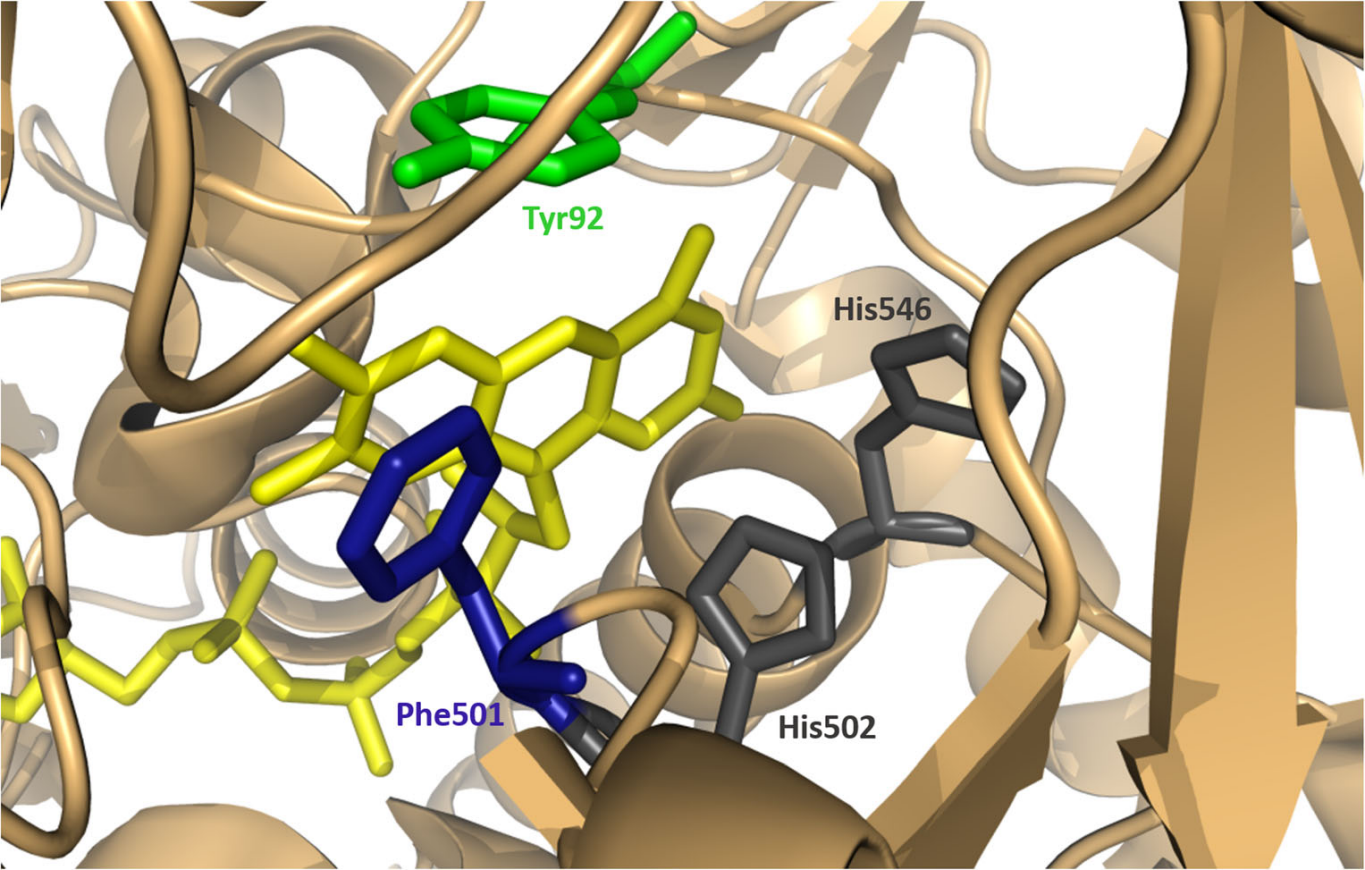
Catalytic site of *Pe*AAO. View of the active site of *Pe*AAO (PDB entry 3FIM) with FAD depicted in yellow, catalytic His502 and His546 depicted in gray, and Tyr92 (green) and Phe501 (blue) located in the hydrophobic substrate access channel

For many years, only the crystal structure of *Pe*AAO from *P. eryngii* with and without inhibitor was available (Carro et al. 2017; Fernandez et al. 2009). The enzyme was produced as selenomethionine derivative in *E. coli* and refolded in vitro. Structure analysis revealed that this AAO is a monomer that is, similar to other GMC oxidoreductases, composed of two domains, an FAD-binding domain and a substrate-binding domain. Non-covalently bound FAD cofactor is stabilized by H bonds formed by the residues of the conserved βαβ fold in the N-terminal domain. In this enzyme, the isoalloxazine ring of FAD is buried inside the protein. The substrate-binding domain is formed by a six-stranded antiparallel β-sheet flanked by two long *α*-helices. Whereas one of the best studied enzymes from the GMC family, glucose oxidase, has an open wide active site, the accessibility to the active site of *Pe*AAO is restricted by an additional loop that forms a funnel-like hydrophobic channel. This channel connects the solvent with the FAD cofactor through a bottleneck formed by three aromatic residues, Tyr92, Phe397, and Phe501, which limit substrate diffusion to the active site. The active site is located in front of the re-side of the isoalloxazine ring of FAD and near two histidines, His502 and His546, postulated as catalytic residues (Ferreira et al. 2006) (Fig. 2).

Sequence comparison of the AAO-PDH clade members of the GMC family revealed that Tyr92 and Phe501 located in the hydrophobic substrate access channel of *Pe*AAO are conserved among many AAOs, but absent in the PDH clade (Sützl et al. 2019). The loss of the narrow hydrophobic channel was suggested as the starting point for widening the substrate entrance site to accept other substrates like sugars by PDH. Another feature to discern AAO and PDH is the covalently bound FAD in PDH. The covalently bound FAD cofactor is usually related to higher redox potentials (Heuts et al. 2009) probably resulting in an altered substrate spectrum and thus contributing to the differentiation of PDHs and AAOs.

In 2020, the crystal structure of *Mt*AAOx from *T. thermophilus* (formerly *Myceliophthora thermophila*) was solved and compared with the structure of *Pe*AAO (Kadowaki et al. 2020). *Mt*AAOx was expressed and secreted by *A. nidulans* and crystallized in glycosylated form. Comparative analysis of the two structures has revealed that whereas the FAD-binding domain is strongly conserved in *Mt*AAOx and *Pe*AAO, the substrate-binding domain and the substrate channel to the active site differ. *Mt*AAOx possesses a wider substrate access channel connecting the active site with the solvent. The conserved catalytic histidines His579 and His622 are located in the easily accessible hydrophobic active site. Remarkably, three aromatic residues in the hydrophobic bottleneck present in *Pe*AAO, Tyr92, Phe397, and Phe501, are not conserved in the *Mt*AAOx structure. Furthermore, the *Mt*AAOx structure has a unique Ca^2+^ binding pocket located near the catalytic site entrance. This element was not reported previously for any AA3_2 enzyme, and seems to stabilize the structure of the thermostable *Mt*AAOx (Kadowaki et al. 2020).

Like other GMC enzymes, the catalytic cycle of AAOs can be divided into two half-reactions, reductive and oxidative. In the reductive half-reaction, the alcohol substrate is oxidized via two-electron abstraction and the reduced form of the flavin cofactor is formed; in the oxidative half-reaction, reduced FAD is reoxidized whereas molecular oxygen is reduced to hydrogen peroxide. Crystal structures, kinetic and spectroscopic analyses, QM/MM studies, docking experiments, and site-directed mutagenesis performed by A. T. Martinez and colleagues have provided a detailed picture of substrate access in the active site and catalytic mechanism of *Pe*AAO which are summarized in the recent review (Serrano et al. 2020). The alcohol substrate interacts with Phe397 in the funnel-like hydrophobic channel, which induces a rearrangement of the loop that limits the access to the active site (Carro et al. 2018a). This rearrangement promotes the substrate in a catalytically relevant position in the active site, where it is stabilized through an aromatic stacking interaction with Tyr92 (Ferreira et al. 2015b). Docking of *p*-methoxybenzyl alcohol and QM/MM calculations demonstrated that the pro-*R* hydrogen of the substrate is located 2.4 A away from the N5 position of the isoalloxazine ring of the cofactor, enabling hydride transfer and yielding the reduced FAD during the reductive half-reaction. In the same time, the hydroxyl proton of the substrate is abstracted by the catalytic base His502, assisted by His546, which forms an H bond with the alcohol (Hernandez-Ortega et al. 2012c). Although proton abstraction and hydride transfer are highly coupled, they occur through non-synchronous concerted mechanism. Unlike other GMC oxidoreductases, no stable alkoxide intermediate was detected, which suggests that AAO follows a different mechanism for alcohol oxidation (Hernandez-Ortega et al. 2011a).

Molecular oxygen accesses the buried FAD by diffusion through the narrow hydrophobic channel, where Phe501 was proposed to assist the O_2_ molecule to approach the flavin C4a position (Hernandez-Ortega et al. 2011b). The structure of *Mt*AAOx from *T. thermophilus* reveals a wider channel, which might explain the 202-times lower O_2_ reactivity of this oxidase than *Pe*AAO. Interestingly, the replacement of Phe501 through alanine in *Pe*AAO resulted in reduction of O_2_ reactivity (Hernandez-Ortega et al. 2011b).

In the oxidative half-reaction in *Pe*AAO, reduced FAD first reacts with molecular oxygen to form a superoxide anion radical and neutral flavin semiquinone. Subsequently, one hydride (originally abstracted from the alcohol substrate in the reductive half-reaction) from the flavin N5 and one proton originating either from the catalytic His502 or from the solvent are transferred to the superoxide anion to furnish H_2_O_2_ (Carro et al. 2018c). The question, whether the first electron transfer is coupled to the proton transfer remains open. Differently from flavin monooxygenases (Paul et al. 2021; Toplak et al. 2021) and some flavin oxidases (Sucharitakul et al. 2008; Wongnate and Chaiyen 2013), a C4a-hydroperoxyflavin is not stabilized in *Pe*AAO (Serrano et al. 2020).

## Substrate spectrum

**Fig. 3:**
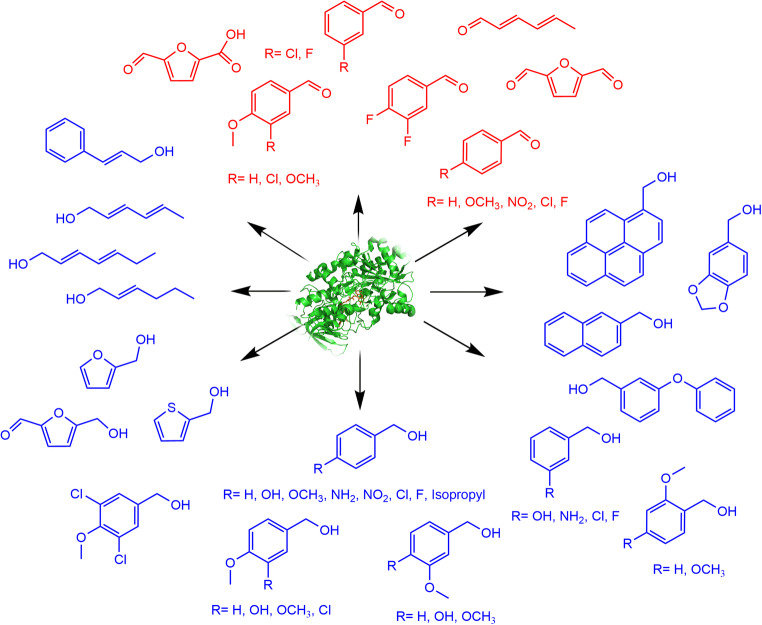
Substrates accepted by AAOs. Alcohols are depicted in blue, aldehydes in red

**Fig. 4:**
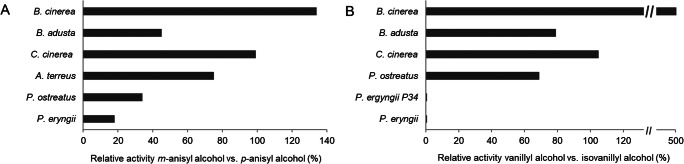
Substrate specificity of AAOs towards *m*- and *p*-anisyl alcohol (**A**) and vanillyl and isovanillyl alcohol (**B**). **A** Relative activity of AAOs from *B. cinerea* (Goetghebeur et al. 1992), *B. adusta* (Romero et al. 2009), *C. cinerea* (Tamaru et al. 2018), *A. terreus* (Chakraborty et al. 2014), *P. ostreatus* (Sannia et al. 1991), and *P. eryngii* (Guillen et al. 1992) towards *m*-anisyl alcohol. Activity towards *p*-anisyl alcohol was set to 100%. **B** Relative activity of AAOs from *B. cinerea* (Goetghebeur et al. 1992), *B. adusta* (Romero et al. 2009), *C. cinerea* (Tamaru et al. 2018), *P. ostreatus* (Sannia et al. 1991), *P. eryngii* P34 (Jankowski et al. 2020), and *P. eryngii* (Guillen et al. 1992) towards vanillyl alcohol. Activity towards isovanillyl alcohol was set to 100%

Originally declared as aryl-alcohol oxidases, the enzymes belonging to this group accept a quite broad range of substrates (Fig. 3). However, substrate preferences differ for different AAOs. Activity of AAOs from *Pleurotus* strains was found to depend on the position and properties of aromatic ring substituting groups (Guillen et al. 1992; Jankowski et al. 2020). Whereas an electron-withdrawing substituent like nitro group at the *para*-position of the aromatic ring had a negative effect on enzyme activity (Guillen et al. 1992), an electron-donating substituent like methoxy-group at this position enhanced enzyme activity compared to unsubstituted benzyl alcohol (Galperin et al. 2016; Guillen et al. 1992; Jankowski et al. 2020; Varela et al. 2000). Other AAOs like r*Cc*AAO from *C. cinerea*, BAO from *B. cinerea*, and AOx from *A. terreus* oxidized benzylic alcohols with a methoxy-substitution at the *para*- or *meta*-position of the aromatic ring equally well (Fig. 4A). Interestingly, the presence of *para*-hydroxy group differently affected the activity of AAOs: In comparison with benzyl alcohol, *p*-hydroxybenzyl alcohol was oxidized with a negligible activity by *Pe*AAO (Guillen et al. 1992), but faster by *C. cinerea* r*Cc*AAO (Tamaru et al. 2018). Whereas vanillyl alcohol was barely oxidized by *Pe*AAO2 from *P. eryngii* P34, isovanillyl alcohol was oxidized by the same enzyme with an almost 3-times higher activity than benzyl alcohol (Jankowski et al. 2020) (Fig. 4B). Differently, *C. cinerea* r*Cc*AAO catalyzed oxidation of both vanillyl alcohol and isovanillyl alcohol with similar activities, which were only slightly lower than activity towards benzyl alcohol (Chakraborty et al. 2014; Tamaru et al. 2018). AAO of *Bjerkandera* BOS55 prefers mono- and dichlorinated anisyl alcohols as substrates (de Jong et al. 1994). Polyunsaturated aliphatic alcohols like *trans*,*trans*-2,4-hexadien-1-ol or 2,4-heptadien-1-ol were recognized as favorable substrates of many AAOs (Guillen et al. 1992; Jankowski et al. 2020; Tamaru et al. 2018). Non-conjugated aliphatic allylic alcohols like *trans*-2-hexen-1-ol, *trans*-2-hepten-1-ol, *trans*-2-octen-1-ol, and *trans*-2-cis-6-nonadien-1-ol were oxidized by *Pe*AAO2 from *P. eryngii* P34 to a much lesser extent (Jankowski et al. 2020). Of the aromatic alcohols, 2-naphthalenemethanol was identified as most easily oxidized by *Pleurotus* AAOs followed by 1-pyrenemethanol (Guillen et al. 1992; Jankowski et al. 2020). 9-Anthracenemethanol was not accepted as substrate at all, probably due to steric constraints. Recently, piperonyl alcohol, cumic alcohol, and 2-thiophenemethanol were identified as AAO substrates as well (Jankowski et al. 2020). These results indicate that the substrate scope of AAOs is dependent on the chemical properties and reactivity of the substrate and is controlled by the size and properties of the active sites. Obviously, a comparative structure-function analysis including more structural information from various AAOs will enable the identification of positions or regions in the active sites responsible for different substrate preferences and provide a basis for rational or semi-rational protein engineering of different AAOs.

In rare cases, AAOs catalyze the oxidation of aldehyde substrates to the corresponding acids. Aldehyde oxidation catalyzed by AAOs was proposed to mainly proceed via the *gem*-diols formed by aldehyde hydration, and thus following the mechanism analogous to alcohol oxidation (Ferreira et al. 2010). Cleavage of one of the *gem*-diol O–H bonds involves the catalytic His502 as a base, and the presence of electron-withdrawing or electron-donating ring substituting groups seems to stabilize or destabilize the *gem*-diol intermediate. In contrast to the substituted aromatic alcohols, *Pe*AAO demonstrated the highest activity towards the aromatic aldehydes with electron-withdrawing substituents like nitro groups or chlorine at the *para*- or the *orth*o-positions. Although benzaldehyde was accepted as substrate, the highest activity of *Pe*AAO was observed with *p*-chlorobenzaldehyde and *p*-nitrobenzaldehyde. The presence of an electron-donating methoxy-group at the *para*-position resulted in low or no activity like with veratraldehyde and *p*-anisaldehyde (Ferreira et al. 2010).

## Heterologous expression

Although identified almost 60 years ago and widespread in nature, only a limited number of AAOs have been characterized so far. Since production levels of AAOs in their original host organisms are usually low and their purification from protein mixtures is tedious, overexpression of AAOs in heterologous microorganisms appears attractive to obtain sufficient amounts of enzymes for purification and biochemical characterization. Significant efforts have been undertaken to achieve high-level expression of AAOs in several microbial hosts, including *Escherichia coli*, *Aspergillus nidulans*, *Pichia pastoris*, *Saccharomyces cerevisiae*, and *C. cinerea* (Table 1). However, their heterologous expression seems to be quite challenging and often suffers from low yields. The first heterologous expression of an AAO was reported in 2001 by Varela and colleagues (Varela et al. 2001). They produced *Pe*AAO in *A. nidulans* using an alcohol dehydrogenase promoter of *A. nidulans* (alcA) with a maximal volumetric activity of 400–500 mU/mL culture. A two-step chromatographic purification procedure yielded 3 mg of active enzyme from 1 L of *A. nidulans* culture (Ferreira et al. 2005). Expression of the *PeAAO* gene without its signal peptide sequence in *E. coli* under the control of the *tac*-promoter resulted in formation of inclusion bodies. After in vitro refolding and purification, 45 mg of active AAO per liter of *E. coli* culture was obtained (Ruiz-Duenas et al. 2006). However, non-glycosylated *Pe*AAO expressed in *E. coli* showed lower pH and temperature stability compared to the glycosylated enzyme isolated from *P. eryngii* or expressed in *A. nidulans* (Ruiz-Duenas et al. 2006). To further improve AAO production level and to establish an appropriate expression host suitable for directed evolution, *S. cerevisiae* was investigated as host for the expression of *Pe*AAO (Vina-Gonzalez et al. 2015). First, different secretion signal peptides of *S. cerevisiae* were investigated to promote secretion of *Pe*AAO. The native secretion signal sequence of *Pe*AAO was replaced by either the prepro-leader sequence from the mating α-factor (preproα) or the prepro-leader sequence from the K_1_ killer toxin (preproK) or chimeras of both, respectively. The preproα-AAO variant was produced at the highest yield of 1.5 U/L followed by the construct with a chimeric leader sequence preαproK-AAO secreted at 0.5 U/L. This difference might be explained by differences in efficiencies of cleavage of the signal peptide sequences. The preproα sequence contains both the KEX2 and STE13 cleavage sites, while the preproK sequence carries only the KEX2 cleavage site. When high amounts of a heterologous protein bearing the α-factor prepro-leader sequence are expressed in *S. cerevisiae*, the amount of STE13 protease in the Golgi might be not enough for efficient cleavage (Ahmad et al. 2014). Since the proK peptide was efficiently cleaved off by KEX2 protease, the preαproK-AAO variant was used to further improve the *Pe*AAO secretion and activity by directed evolution. Several *Pe*AAO mutant libraries were constructed by conventional directed evolution and using the method named Mutagenic Organized Recombination Process by Homologous In Vivo Grouping (MORPHING) in *S. cerevisiae* (Vina-Gonzalez et al. 2015). Out of 5000 clones screened, the FX7 variant with the mutation H91N demonstrated a 96-fold improved total activity compared to the parental preαproK-AAO variant. FX7 was expressed at a concentration of 2 mg/L as a heavily glycosylated protein (~50% glycosylation) and possessed biochemical properties comparable to those of *Pe*AAO expressed in *E. coli*, while pH and thermal stabilities were significantly enhanced. To further improve *Pe*AAO secretion in *S. cerevisiae*, in vivo shuffling of the FX7 variant with six other improved variants was combined with subsequent MORPHING of the preαproK leader sequence (Vina-Gonzalez et al. 2018). The most active clone FX9 with 4 mutations in the preαproK peptide and 2 mutations, H91N and L170M, in the mature protein was secreted at concentration of 4.5 mg/L during cultivations in shake flasks. Expression of the FX9 variant in *P. pastoris* under control of the methanol-inducible *AOX1* promoter yielded 25.5 mg/L of enzyme (final activity of 1378 U/L) from a 5 L fed-batch culture (Vina-Gonzalez et al. 2018). The *P. pastoris*–expressed FX9 variant showed different kinetic parameters and lower thermal stability than its counterpart expressed in *S. cerevisiae*. These differences were attributed to the poor glycosylation of the FX9 variant expressed in *P. pastoris* (degree of glycosylation not measurable) compared to heavy glycosylation when expressed in *S. cerevisiae* (60% glycosylation). Another *Pleurotus* AAO from *P. sapidus* was heterologously expressed in *C. cinerea* under control of the *Agaricus bisporus gpdII* promoter up to a volumetric activity of 125 U/L (Galperin et al. 2016). Recently, heterologous expression of *Pe*AAO2 from *P. eryngii* P34 in *P. pastoris* was reported with an exceptional high yield of 315 mg/L and a volumetric activity of 7250 U/L after fed-batch cultivation in 3 L (Jankowski et al. 2020). Although mature *Pe*AAO2 and the FX9 variant of *Pe*AAO differ only in 9 amino acid residues located on or near the protein’s surface, *Pe*AAO2 was much better expressed than FX9 and more stable at acidic and basic pH values. The higher pH stability might be due to glycosylation, since *Pe*AAO2 was strongly glycosylated (30% *N*-glycosylation) in *P. pastoris*. Two further AAOs, *Um*AAO from *Ustilago maydis* and r*Cc*AAO from *C. cinerea*, were also expressed in *P. pastoris* (Couturier et al. 2016; Tamaru et al. 2018). *Um*AAO was secreted with the help of the α-factor prepro-leader peptide and expressed with a C-terminal (His)_6_-tag. Around 1 g of recombinant *Um*AAO per liter of culture medium was produced which is the highest yield of an AAO reported so far. r*Cc*AAO was also fused to the α-factor prepro-leader peptide for secretion, but unfortunately no information on expression yields were provided. Kadowaki and coworkers reported on heterologous expression of *Mt*AAOx from *T. thermophilus* M77 in *A. nidulans* (Kadowaki et al. 2020). The *Mt*AAOx gene was expressed using a glucoamylase promoter and a heterologous signal peptide. After three chromatographic steps, 8.4 mg of *Mt*AAOx was obtained from 1 L of culture. The group of Wilkins reported on heterologous expression of an AAO from *T. thermophilus* in *A. nidulans* as well (Liu et al. 2020; Pardo-Planas et al. 2017). Process optimization and up-scaling of protein production to 3-L stirred-tank bioreactor resulted in volumetric activity of 1906 U/L after 48 h (Liu and Wilkins 2020). However, no sequence information of the enzyme was provided and activity of this enzyme towards veratryl alcohol was solely measured with 2,6-dichlorophenol indophenol (DCPIP) as final electron acceptor. The question if this enzyme is identical or highly similar to *Mt*AAOx remains open.

**Table 1:** Heterologous expression of AAOs in different hosts

Name	Origin	Expression host	Vol. Activity (U/L)	Yield (mg/L)	Reference
*Pe*AAO	*Pleurotus eryngii*	*Aspergillus nidulans*	−	3^a^	Ferreira et al. (2005)
*Pe*AAO	*Pleurotus eryngii*	*Aspergillus nidulans*	400-500	−	Varela et al. (2001)
*Pe*AAO	*Pleurotus eryngii*	*Escherichia coli*	−	45^b^	Ruiz-Duenas et al. (2006)
*Pe*AAO_FX7	*Pleurotus eryngii*	*Saccharomyces cerevisiae*	48	2	Vina-Gonzalez et al. (2015)
*Pe*AAO_FX9	*Pleurotus eryngii*	*Saccharomyces cerevisiae*	−	4.5	Vina-Gonzalez et al. (2018)
*Pe*AAO_FX9	*Pleurotus eryngii*	*Pichia pastoris*	1378	25.5	Vina-Gonzalez et al. (2018)
*Pe*AAO2	*Pleurotus eryngii* P34	*Pichia pastoris*	7250	315	Jankowski et al. (2020)
AAO	*Pleurotus sapidus*	*Coprinopsis cinerea*	125	−	Galperin et al. (2016)
rAOx	*Aspergillus terreus* MTCC6324	*Escherichia coli*	−	−	Chakraborty et al. (2014)
r*Cc*AAO	*Coprinopsis cinerea*	*Pichia pastoris*	−	−	Tamaru et al. (2018)
*Mt*AAOx	*Thermothelomyces thermophilus* M77	*Aspergillus nidulans*	−	8.4	Kadowaki et al. (2020)
AAO	*Thermothelomyces thermophilus*	*Aspergillus nidulans*	1906^c^	−	Liu and Wilkins (2020)
*Um*AAO	*Ustilago maydis*	*Pichia pastoris*	−	1000	Couturier et al. (2016)

^a^Purified enzyme

^b^Purified and refolded from inclusion bodies

^c^Activity measured with DCPIP as final electron acceptor

The second AAO heterologously expressed in *E. coli* is rAOx from *A. terreus* MTCC6324 (Chakraborty et al. 2014). rAOx expressed with both N- and C-terminal (His)_6_-tag under control of the T7 promoter in *E. coli* BL21(DE3) predominantly formed inclusion bodies. After purification and in vitro refolding of aporAOx with its cofactor FAD, active rAOx was obtained, but no information on achieved yields were provided.

## Biotechnological application

**Fig. 5:**
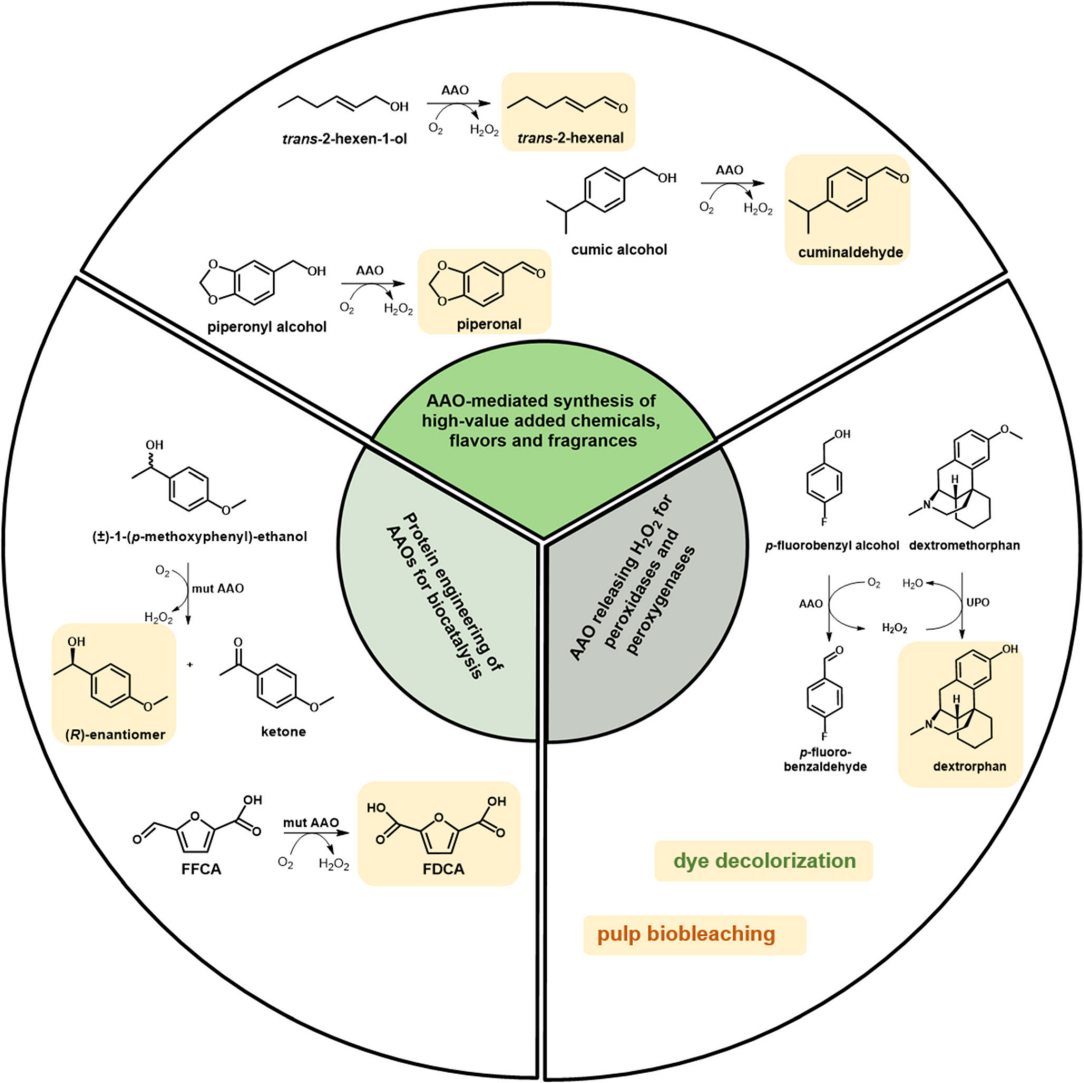
AAOs for biotechnological purposes

The ability of AAOs to reduce molecular oxygen to hydrogen peroxide coupled to alcohol oxidation makes them interesting candidates for biotechnological purposes (Fig. 5). On the one hand, AAOs releasing H_2_O_2_ are used to support reactions catalyzed by peroxidases and peroxygenases. On the other hand, AAO-catalyzed oxidation reactions provide a valuable tool for the synthesis of building blocks for chemical synthesis and flavors. Finally, there are processes in which AAOs are applied for both, biocatalytic oxidation and for in situ H_2_O_2_ formation to support other enzymes involved in multi-enzyme cascades for the production of bio-based precursors for polymers. Furthermore, using methods of protein engineering AAOs was optimized for application as effective biocatalysts. In the following, we will give a short overview of the processes and reactions involving AAOs.

### Dye decolorization and pulp biobleaching

AAOs lead to H_2_O_2_ production and hence can support various enzymes during dye decolorization and lignin degradation. Synthetic dyes are widely used in the paper and textile industry causing large amounts of contaminated wastewater. Efficient wastewater treatment is thus of great importance to remove these harmful compounds being toxic to humans and aquatic organisms (Ledakowicz and Pazdzior 2021). Besides physical and chemical treatments, biological methods relying on fungi, bacteria, or isolated enzymes are of high importance. Among others, fungal ligninolytic enzymes, such as laccases, LiP, and MnP, have been broadly used for dye decolorization (Wesenberg et al. 2003). Only one report describes the direct participation of AAO in dye decolorization: AAO isolated from *Sphingobacterium* sp. ATM caused the degradation of the textile dyes Red 5B, Orange 3R, and Direct Blue GLL within 8 h (Tamboli et al. 2011). Other investigations revealed that AAOs contribute to the decolorization process by generating H_2_O_2_ for the dye degrading enzymes. For example, AAO from *Geotrichum candidum* Decl was not able to degrade textile dyes like Red 5B, but supported its decolorization by delivering H_2_O_2_ to the dye peroxidase DyP. Moreover, AAO was suggested to prevent the polymerization of dye products produced by DyP peroxidase, which facilitated their further degradation (Kim et al. 2001). A combination of dye decolorizing peroxidase DyP from *Mycetinis scorodonius* and AAO from *P. sapidus* resulted in a more efficient decolorization of the dye Reactive Blue 5 in comparison to the DyP directly supplied with external H_2_O_2_. This effect was attributed to the low but constant production of H_2_O_2_ by AAO, which allows to overcome the possible DyP inhibition by high H_2_O_2_ concentrations (Galperin et al. 2016). This two-enzyme system does not require additional equipment and online quantification of H_2_O_2_ concentration and thus has a potential to replace the systems for in situ H_2_O_2_ supply like feeding or electrochemical peroxide production.

AAOs have been shown to contribute also to the pulp bleaching process to obtain white paper pulp. Original pulp color is impacted by the chromophores of lignin. Thus, a set of hydrolases and oxidoreductases involved in lignin degradation have been tested for removal of these chromophores and pulp lightening. Whereas *P. cinnabarinus* laccase combined with the redox mediator 1-hydroxybenzotriazol (HBT) demonstrated the strongest delignification of pulp, the same laccase without mediator also caused pulp lightening that was increased in the presence of AAO (Sigoillot et al. 2005). Similar to the effect on dye decolorization, the positive effect of AAO was attributed to their ability to prevent repolymerization of the lignin phenoxy radicals produced by laccase (Sigoillot et al. 2005).

### Synthesis of flavors and valuable building blocks

Flavors and fragrances are widely used in food and beverages, as well as in the cosmetic, detergent, and pharmaceutical industries. They are currently mainly produced by chemical synthesis. Due to the increasing consumer demand for “natural” flavors, more and more biotechnological processes relying on enzymes or microorganisms are established for the production of these compounds. Vanillin and benzaldehyde (cherry flavor), the leading compounds in the flavor and fragrance industry, can be formed, for example, via AAO-catalyzed oxidation of the corresponding alcohols, which makes AAOs interesting candidates for the synthesis of flavors and fragrances. For instance, recombinant *Pe*AAO from *P. eryngii* catalyzes the oxidation of *trans*-2-hexen-1-ol to *trans*-2-hexenal, a major component responsible for the fresh flavor of fruits and vegetables. *trans*-2-Hexenal is widely applied in the flavor and fragrance industry as Green Note in food, beverages, and perfumes. In an attempt to enhance the biocatalytic performance of *Pe*AAO that can be limited by the poor solubility and low diffusion rates of O_2_ into the aqueous reaction media, the reaction was conducted in a continuous slug-flow microreactor (van Schie et al. 2018). Additionally, catalase was added to decompose the produced H_2_O_2_ to H_2_O and O_2_. With this setup, 10 mM *trans*-2-hexen-1-ol were completely converted to *trans*-2-hexenal within the residence time of 40 min, which corresponds to a turnover number (TN) of 32,400. By increasing residence time in a longer flow reactor, TNs of over 300,000 were reached (van Schie et al. 2018). Further increase of substrate concentration was however limited by low solubility of *trans*-2-hexen-1-ol in aqueous media. The use of two liquid-phase systems allowed not only to increase substrate solubility but also to avoid product inhibition (de Almeida et al. 2019). Dodecane and *trans*-2-hexen-1-ol itself as organic phase ensured the best results among the organic solvents tested. At a 1:1 ratio of aqueous to dodecane phase, full conversion of 500 mM *trans*-2-hexen-1-ol was achieved after 24 h. This corresponds to a TN of 650,000. When *trans*-2-hexen-1-ol was used as organic phase at a 4:1 ratio and both, *Pe*AAO and catalase, were added six times in course of the reaction, 2.6 M *trans*-2-hexenal were produced within 14 days, which corresponded to a TN of 2,200,000. These examples demonstrate high robustness of the recombinant *Pe*AAO from *P. eryngii*. In another study, *Pe*AAO2 from *P. eryngii* P34 was shown to catalyze the oxidation of piperonyl alcohol to piperonal with the second highest catalytic efficiency among the substrates investigated with this enzyme (Fig. 5) (Jankowski et al. 2020). Piperonal, also termed as “heliotropin,” has a sweet, floral odor and is used in the perfumery and cosmetic industry, but also in the food industry as vanilla aroma. Piperonal is also used as an intermediate in the production of insecticides, pesticides, and pharmaceutical agents for, e.g., treatment of Alzheimer’s disease (Brum et al. 2019; Santos et al. 2004; Wang et al. 2019).

AAOs are also of potential interest for the synthesis of chiral secondary alcohols. Docking of *p*-methoxybenzyl alcohol in the active site of *Pe*AAO has revealed a stereoselective hydride abstraction from the pro-*R* Cα position (Hernandez-Ortega et al. 2012b). The stereoselective hydride abstraction from the *S*-enantiomer would lead to the corresponding ketone, while the *R*-enantiomer would remain unaffected. Chiral secondary alcohols are essential building blocks for the production of, e.g., pharmaceutical compounds. However, activity of *Pe*AAO with secondary aromatic alcohols is much lower than with primary alcohols probably due to the narrow substrate access channel. To enhance activity of *Pe*AAO towards secondary alcohols, rational protein design and directed evolution approaches have been applied (Serrano et al. 2019b; Vina-Gonzalez et al. 2019). The *Pe*AAO variant F501A, where phenylalanine limiting substrate diffusion was replaced by the smaller alanine (Hernandez-Ortega et al. 2012b), was used as a starting enzyme for adaptive-protein energy landscape exploration simulations and combinatorial saturation mutagenesis (Serrano et al. 2019b). As a result, the double mutant I500M/F501W with increased activity and stereoselectivity was identified. Introducing the mutations I500M and F501W into the *Pe*AAO variant FX9 with improved secretion (see above), followed by structure-guided evolution including in vivo site-directed recombination as the final step, resulted in the LanDo variant with a more than 800-fold enhanced activity towards 1-(*p*-methoxyphenyl)-ethanol compared to FX9 (Vina-Gonzalez et al. 2019). The catalytic efficiency (*k*_cat_/*K*_m_) of the LanDo variant with 1-(*p*-methoxyphenyl)-ethanol was 10-fold higher than of the I500M/F501W variant. The *S*-enantiomer of racemic 1-(*p*-methoxyphenyl)-ethanol was almost completely oxidized by the LanDo variant, which resulted after 2 h in *R*-enantiomer with *ee* >99%. This example demonstrates the potential of AAOs as biocatalyst for the synthesis of chiral secondary alcohols via deracemization.

Another potential field of AAO application lies in the production of compounds with beneficial effects on human health or drug metabolites. *Pe*AAO2 from *P. eryngii* P34 catalyzes the oxidation of cumic alcohol to cuminaldehyde with the highest activity among the substrates tested with this enzyme. Cuminaldehyde is the major constituent of seed oil of *Cuminum cyminum* (Lee 2005; Li and Jiang 2004) with potential therapeutic effects (Morshedi et al. 2015). For example, cuminaldehyde demonstrated anticancer and antidiabetic properties (Lee 2005; Patil et al. 2013; Tsai et al. 2016).

Recently, a self-sufficient unspecific peroxygenase (UPO)/aryl-alcohol oxidase biocatalyst for the synthesis of dextrorphan, a human metabolite of the antitussive drug dextromethorphan, was reported (de Santos et al. 2020). The bifunctional fusion consisted of a UPO catalyzing the transformation of dextromethorphan to dextrorphan and an AAO supplying UPO with H_2_O_2_ through the oxidation of primary aromatic alcohols. The engineered SoLo variant of UPO from *Agrocybe aegerita* with reduced peroxidase activity towards aromatic alcohols and the *Pe*AAO variant FX9 were chosen as fusion partners. At first, several enzyme fusion libraries were constructed consisting of fusions with different partner order and rigid or flexible linkers of different length and compositions between UPO and AAO. For secretion of the fusion construct in *S. cerevisiae*, two evolved leader sequences were tested, the preαproK leader peptide for AAO and an evolved signal peptide for UPO. Five UPO-AAO fusions with the highest activities were purified and biochemically characterized. To exclude unwanted interactions of AAO substrates with the UPO partner, several primary aromatic alcohols were tested, and *p*-fluorobenzyl alcohol, 3-methoxybenzyl alcohol, and *p*-methoxybenzyl alcohol led to the best results. By using UPO-AAO fusion with *p*-fluorobenzyl alcohol as sacrificial substrate, 2 mM dextrorphan was obtained from 2 mM dextromethorphan after 24 h, and a TTN of 48,300 was achieved. Since oxidation of *p*-fluorobenzyl alcohol proceeded much faster than dextromethorphan hydroxylation, accumulated H_2_O_2_ most likely inactivated the UPO partner. Feeding of *p*-fluorobenzyl alcohol at 0.5 mM/h resulted in increased TTN for the synthesis of dextrorphan of over 62,000. The construction of UPO-AAO fusions for the synthesis of pharmaceutical or chemical compounds further expands the scope of application of AAOs.

### Production of bio-based precursors for polymers

**Fig. 6:**
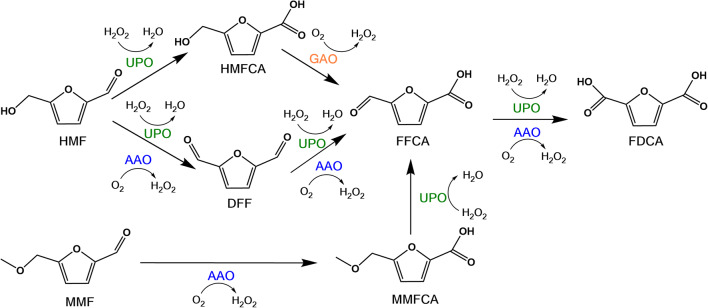
Enzymatic oxidation of HMF and MMF to FDCA involving AAO

In times of an emerging bioeconomy, the sustainable production of bio-based polymer precursors or platform chemicals becomes more and more important. The use of bio-based 2,5-furandicarboxylic acid (FDCA) for the production of the biopolymer polyethylene furanoate (PEF), which could replace polyethylene terephthalate (PET), strongly contributes to establishing a sustainable bioeconomy. The chemical synthesis of FDCA takes place at high temperature and pressure, and involves organic solvents and metal catalysts (Sajid et al. 2018). The oxidation of 5-hydroxymethylfurfural (5-HMF) to FDCA proceeds via 2,5-diformylfuran (DFF) and 5-formylfurancarboxylic acid (FFCA). Enzymatic routes from 5-HMF to FDCA, involving, for example, AAO (Fig. 6), can be conducted at ambient temperature without the need of polluting co-solvents. 5-HMF can be obtained by acid-catalyzed dehydration of fructose. Dijkman and Fraaije (2014) described a HMF oxidase (HMFO) from *Methylovorus* sp. strain MP688, a member of the GMC oxidoreductase family with AAO activity, which was expressed in *E. coli* and oxidized 5-HMF to FFCA as the main product (92%), but only minor amounts of FDCA (8%) were formed after 5 h of reaction. Recently, two other HMFOs from *P. nitroreducens* and *Pseudomonas* sp. strain 11/12A, both heterologously expressed in *E. coli*, were shown to catalyze oxidation of 5-HMF to up to 99% FDCA after 96 h (Vinambres et al. 2020). Carro and coworkers found that *Pe*AAO expressed in *E. coli* also catalyzed the conversion of 5-HMF to FFCA, but no subsequent oxidation of FFCA to FDCA was detected. To complete the second reaction, an unspecific peroxygenase (UPO) from *A. aegerita* was added after oxidation of 5-HMF to FFCA yielding 91% FDCA after 120 h. Another approach was based on the combined action of three fungal enzymes, AAO, UPO, and galactose oxidase (GAO), for the conversion of 5-HMF to FDCA (Karich et al. 2018). AAO catalyzed the oxidation of 5-HMF to DFF along with the production of H_2_O_2_ to be used by UPO for the oxidation of 5-HMF to 5-hydroxymethyl-2-furancarboxylic acid (HMFCA) and to FDCA via the intermediates DFF and FFCA. GAO provided additional H_2_O_2_ for UPO while oxidizing 5-HMF to DFF and particularly HMFCA to FFCA. Starting with 10 mM 5-HMF, 7.9 mM FDCA was formed in this three-enzyme approach after 24 h. Serrano and colleagues recently reported that *Pe*AAO expressed in *E. coli* catalyzes also the oxidation of FFCA to FDCA, with a 100-fold lower reaction rate compared to 5-HMF or DFF oxidation (Serrano et al. 2019a). They found that the oxidation of FFCA was negatively affected by the presence of H_2_O_2_ particularly at concentrations over 400 μM, although the enzyme was quite stable in the presence of peroxide. By applying catalase together with *Pe*AAO, 5-HMF was completely oxidized to FDCA after 12 days, which confirmed the inhibitory effect of H_2_O_2_ on FFCA oxidation. When using *Pe*AAO variant I500M/F501W in catalase-assisted conversion of 15 mM 5-HMF to FDCA, a nearly 2-fold increase in total turnover number (TTN) was achieved.

Vina-Gonzalez and colleagues further engineered *Pe*AAO for improved conversion of 5-HMF to FDCA (Vina-Gonzalez et al. 2020). Combinatorial saturation mutagenesis of the variant FX9 at positions involved in substrate positioning in the active site, Ile500 and Phe501 in the first round, and Tyr92 and His546 in the second round, leading to the Bantha variant with the additional mutation F501W as the most active enzyme. The catalytic efficiency of the Bantha variant was 3-fold higher compared to *Pe*AAO wild-type. Formation of FDCA was 6-fold higher when using the Bantha variant and reached 3.0% after 2 days of reaction starting from 2 mM 5-HMF as substrate.

An alternative route for the production of FDCA is based on 5-methoxymethylfurfural (5-MMF) as starting compound, which is more stable upon storage than 5-HMF. For this route, another three-enzyme system was chosen that consisted of AAO, UPO, and methanol oxidase (MOX) (Carro et al. 2018b). AAO catalyzed the oxidation of 5-MMF to MMFA, while UPO catalyzed the two-step oxidation of MMFA to FDCA via the intermediate FFCA. The activity of UPO was supported by H_2_O_2_ generated by AAO and additionally in the MOX-mediated oxidation of methanol to formaldehyde. After 120 h, 98% FDCA were formed starting with 1.5 mM 5-MMF. These examples illustrate the usefulness of AAOs for valorization of products from lignocellulose biorefinery.

## Concluding remarks and future perspectives

More than 60 years after the discovery of AAO in *T. versicolor*, a large number of AAO encoding genes were identified in the genomes of many wood-decaying fungi. Their functions in nature and the general catalytic mechanism are considered deciphered, and the reactions catalyzed by AAOs and summarized here impressively demonstrate the high potential of these enzymes for biotechnological purposes. Advances in protein engineering have prompted the construction of AAO variants with altered substrate scope and increased stereoselectivity, which opens new perspectives for synthetic chemistry. Realization of AAO-catalyzed reactions in two-phase systems and in a flow reactor illustrates the high robustness of these biocatalysts and their potential applicability in technical processes at high scale.

However, structural and mechanistic aspects have been tackled mainly based on the crystal structure and molecular modelling of one representative - *Pe*AAO from *P. eryngii*. In most cases, this enzyme and mutants thereof were successfully used for the synthesis of valuable compounds. So far, only a few other AAOs were purified and characterized in respect to their biochemical properties and substrate spectra. Since AAO sequences demonstrate low identity, three-dimensional structures of other members would provide more insights into catalysis and substrate recognition, and allow to rationalize differences in substrate spectra and properties of these enzymes. In 2020, the second structure of an AAO originating from *T. thermophilus* has been solved, which revealed several significant differences compared to the structure of *Pe*AAO. Structural information on other AAOs and their comparison could build a broader background for enzyme engineering to promote recognition and oxidation of industrially relevant compounds with high activity and selectivity. However, heterologous expression of AAOs in recombinant hosts remains a challenging task and seems to limit their broader application and protein engineering. Although significant efforts have been undertaken to enhance heterologous expression of AAOs, no straightforward strategy has been established yet. Engineering of the signal peptide facilitated protein secretion of *Pe*AAO in *S. cerevisiae* in the culture medium, but also mutations in the protein sequence positively influenced gene expression (Vina-Gonzalez et al. 2015, 2018). Another intriguing question is why expression of *Pe*AAO in *P. pastoris* failed, whereas the homologous *Pe*AAO2 with only seven different amino acids was expressed at a concentration of 315 mg/L. Obviously, more deep understanding of the effect of these amino acids on transcription, translation, protein folding, secretion, and stability is required and will remain in focus of future studies.

The scope of AAO applications can be expanded by their exploiting as supplier of H_2_O_2_ for various peroxide-dependent enzymes. A contribution to enzyme-mediated delignification of plant biomass in biorefinery concepts seems plausible since ligninolytic peroxidases relying on H_2_O_2_ play a major role in lignin degradation in nature. Along with their use in biobleaching and biopulping in the pulp and paper industry, AAOs might also support peroxidases in polymer synthesis, bioremediation of phenolic compounds, peroxidase-based biosensors, and biocatalysis (Regalado et al. 2004). Reaction engineering will focus on developing substrate-feeding strategies to control the level of H_2_O_2_ released by AAOs in order to avoid its negative effect on heme-containing peroxidases and peroxygenases. Combinations of AAOs with other biocatalysts in multi-step cascade reactions offer alternative routes for the synthesis of various molecules and provide exciting future perspectives to access novel compounds that cannot be synthesized by classical chemical routes.

Thus, the concepts described in this review build a solid background for further development of AAOs towards applications with efficiencies approaching economic viability.

## Data Availability

All data on which the conclusions were drawn are presented in this study.
